# Preparedness and experiences of healthcare professionals during the Hajj mass gatherings: a qualitative study

**DOI:** 10.1186/s12913-026-14390-9

**Published:** 2026-03-17

**Authors:** Thawab Alrabie, Michael Brown, Billiejoan Rice, Lynne Marsh

**Affiliations:** 1 School of Nursing and Midwifery, Queen’s University Belfast, Belfast, UK; 2 College of Nursing, Umm Al-Qura University, Makkah al Mukarramah, Saudi Arabia

**Keywords:** Hajj mass gatherings, Disaster preparedness, Healthcare professionals, Clinical experiences

## Abstract

**Background:**

The Hajj pilgrimage in Makkah, Saudi Arabia, is one of the world’s largest mass gatherings, posing unique challenges to healthcare systems. Healthcare professionals must provide care amid high patient volumes, diverse medical needs, and potential emergencies. Despite growing attention to preparedness, little research has explored the experiences and educational needs of frontline healthcare professionals in this demanding setting.

**Aim:**

To explore the experiences and preparedness of healthcare professionals who participated in the Hajj, with a focus on disaster response, training, and system-level challenges.

**Methods:**

A qualitative exploratory study was conducted using semi-structured interviews with 17 participants, comprising nursing educators, paramedics, and physicians. Participants were purposively sampled and interviewed between January and June 2024. Data were analysed using Reflexive Thematic Analysis to identify patterns and insights related to disaster preparedness and clinical practice during the Hajj.

**Findings:**

Four overarching themes emerged: limited disaster preparedness, a lack of communication and leadership, challenges in accessing prehospital care and patient management, and the need for cultural competence and workforce resilience.

**Conclusion:**

This study identified a lack in disaster training and education, and staff deployment. A systems-based approach and ongoing scenario-based training are essential to improve resilience and inform policy and education for future mass gatherings.

**Supplementary Information:**

The online version contains supplementary material available at 10.1186/s12913-026-14390-9.

## Introduction

Makkah, in the Kingdom of Saudi Arabia, serves as the spiritual epicentre for millions of Muslims worldwide and hosts the annual Hajj pilgrimage, which is one of the largest and most significant mass gatherings on the planet [1]. Each year, more than two million pilgrims converge on the city to perform religious rituals over a concentrated period. While the event is deeply rooted in spiritual devotion, its scale, density, and diversity create significant public health challenges, including risks of heatstroke, crowd injuries, and infectious diseases [2].

The concentration of vast numbers of individuals, often elderly or with underlying health conditions, within confined and physically demanding settings increases the likelihood of a wide range of health emergencies. These include heat-related illnesses, infectious disease outbreaks, crowd crushes, environmental hazards, and infrastructure failures [3]. Historical incidents, such as the 1997 Mina tent fire and the 2015 stampede, further illustrate the range and severity of these risks, highlighting the Hajj as a disaster-prone context in which healthcare professionals must not only deliver routine care but also respond effectively to large-scale, high-pressure emergencies. Consequently, ensuring adequate preparedness through training, education, and structured support systems is essential for safeguarding public health and minimising mortality and morbidity during the pilgrimage [2].

Within this context of recurrent large-scale risks and complex healthcare demands, the Saudi healthcare system implements extensive preparedness measures during the Hajj season to manage the anticipated surge in healthcare needs. These measures include the deployment of temporary hospitals and mobile medical units, the establishment of mini care centres across pilgrimage sites such as Mina, Arafat, and Muzdalifah [2], and the mobilisation of additional healthcare professionals through adjusted leave policies and redeployment strategies. Emergency medical services are reinforced, and coordination is maintained across multiple agencies, including the Ministry of Health, the Saudi Red Crescent Authority, and hospital-based services, to ensure continuity of care throughout the pilgrimage [4].

Despite these efforts, previous studies have identified persistent gaps in disaster preparedness among healthcare professionals involved in Hajj. Alzahrani and Kyratsis reported that more than half of emergency room nurses had never reviewed their disaster response plans, with approximately 10% unaware of the existence of such protocols, indicating shortcomings in institutional preparedness [5]. Similarly, Al-Otaibi found that while paramedics generally demonstrated solid disaster readiness, physicians exhibited a stronger understanding of emergencies specific to the Hajj context, and preparedness levels varied by age and educational background [6].

Further evidence by Al Wathinani demonstrated that older, better-educated EMS personnel, as well as those affiliated with the military, performed more effectively during disaster situations, particularly when they had participated in regular workshops and hands-on training. These findings emphasise the importance of experiential learning in strengthening disaster preparedness [7]. This is further supported by a recent systematic review by Alrabie et al. (2025), which found that healthcare professionals involved in the Hajj exhibited overall low levels of disaster preparedness, with significant gaps in Hajj-specific training, institutional support, and practical experience [8].

In response to these identified gaps, this study explored the experiences and disaster preparedness of healthcare professionals during Hajj, with a particular focus on training, response coordination, and system-level enablers and barriers. The study adopted Systems Theory, which conceptualises preparedness as the outcome of interdependent relationships between individuals, teams, institutions, and policy environments. This framework is particularly well suited to the Hajj context, where multiple actors must coordinate under intense pressure, and it enables the identification of systemic strengths and weaknesses that influence resilience and response effectiveness in mass gathering settings [26].

## Methods

### Study design

This study adopted a qualitative exploratory design to explore the experiences, roles, and perspectives of nursing educators, paramedics, and physicians in the Hajj mass gatherings. This design was selected to allow for an in-depth exploration of the complex and context-specific challenges experienced by healthcare professionals during mass gathering events [9].

### Setting

The study was conducted in Makkah, Saudi Arabia, and reflects healthcare professionals’ experiences across both hospital-based services and pilgrimage-specific healthcare settings operating during the Hajj season. Participants were involved in providing care within temporary medical facilities, emergency response services, and hospitals supporting the pilgrimage.

### Sampling and participants

A purposive sampling strategy was employed to recruit participants with direct clinical experience in the Hajj mass gatherings. The final sample consisted of seventeen participants: six nursing educators, who are clinical educators responsible for training and supervising staff in clinical settings; five physicians, who are practising clinicians directly involved in patient care during the Hajj; and six paramedics, who served as frontline responders. Sample size was guided by data saturation, defined as the point at which no new themes or insights emerged from successive interviews. Saturation was assessed through ongoing preliminary analysis conducted alongside data collection.

### Data collection

Data were collected through individual semi-structured interviews conducted with the lead author, conducted in either Arabic or English, based on participant preference. An open-ended interview guide (see Appendix A) was used to explore participants’ roles, training, experiences, challenges, and recommendations related to disaster response during the Hajj. The guide was developed by the researcher, reviewed for face and content validity by three nursing academics, and piloted with five registered nurses for clarity; no changes were identified. Interviews lasted about 40 min and took place in private settings chosen by participants. With consent, all interviews were conducted in either Arabic or English, according to participant preference. Arabic interviews were transcribed verbatim and subsequently translated into English by the lead author, who is fluent in both languages. Translations were checked for accuracy and meaning consistency prior to analysis.

### Data analysis

Data were analysed using reflexive thematic analysis (RTA) following Braun and Clarke’s six-phase approach. Analysis was inductive and data-driven, with themes generated through immersion in participants’ narratives rather than predetermined theoretical categories [10].

Preliminary analysis commenced after the first six interviews, enabling early familiarisation with the data and the development of initial codes. Coding and reflexive memo-writing continued iteratively alongside data collection. Data saturation was reached after approximately 14 interviews, at which point no new patterns or meanings were identified. Three additional interviews were conducted to confirm saturation, resulting in a final sample of 17 participants.

Codes were developed through careful reading of transcripts and were progressively refined, clustered, and organised into themes that captured shared patterns of meaning across the dataset. Regular reflexive discussions among the research team supported analytic depth and coherence, with differences in interpretation resolved through dialogue rather than consensus coding, consistent with reflexive thematic analysis.

Systems Theory was not used to guide coding or theme development. Instead, it was applied at the interpretive stage to help explain relationships between themes and situate findings within the broader healthcare system operating during the Hajj.

## Findings

This study included 17 participants: six nursing educators, five physicians, and six paramedics. A total of 68 individuals were invited (23 educators, 29 paramedics, and 16 physicians), but due to scheduling conflicts and availability, 17 completed the study. Table 1 summarises participant demographics.

**Table 1:** Participant data

Participant	Role	Age	Gender	Years of Experience	Clinical Area	Hajj Participation (Times)
P1	Educator	35	Female	13	Education	13
P2	Educator	29	Female	4	Intensive care	3
P3	Educator	32	Female	15	Education	3
P4	Educator	39	Male	19	Education	5
P5	Educator	34	Male	10	Education	7
P6	Educator	33	Male	9	Education	5
P7	Physician	35	Female	10	Burn Unit	3
P8	Physician	45	Male	20	Intensive care	5
P9	Physician	49	Male	18	Emergency	7
P10	Physician	46	Female	20	Emergency	5
P11	Physician	47	Male	19	Intensive care	6
P12	Paramedic	26	Male	3	Paramedic	2
P13	Paramedic	35	Male	14	Paramedic	12
P14	Paramedic	37	Male	13	Paramedic	13
P15	Paramedic	28	Male	12	Paramedic	7
P16	Paramedic	25	Male	4	Paramedic	4
P17	Paramedic	29	Male	5	Paramedic	3

### Main findings

The study identified five key themes from interviews with healthcare professionals involved in providing care during the Hajj mass gatherings. These themes represent central aspects of their professional experiences in this complex, high-pressure environment. They address critical areas including overall participation in Hajj, the adequacy of education and training, patient care and management, and the broader preparedness required for large-scale emergency events.

## Hajj preparedness

This major theme explores how healthcare professionals are trained and prepared to participate in the Hajj, particularly their familiarity with relevant policies, procedures, and protocols.

Nursing participants widely reported gaps in disaster preparedness, noting limited knowledge of different types of disasters, such as chemical disasters, and uncertainty about how to respond or which protocols to follow. This lack of awareness was viewed as a significant obstacle to effective disaster response. As one participant stated:

*My knowledge needs more improvement on different scenarios of disasters to ensure preparedness and proper response in times of need (P1).*

Building on this concern, nursing participants noted that although numerous focus sessions and disaster-training activities are offered before Hajj, the timing and delivery of these sessions alone are not enough to ensure true preparedness. They pointed out that skills and knowledge can fade over time, especially if not regularly practised. One participant noted:

*Taking a course at a particular time does not guarantee readiness; it’s important to continuously refresh and maintain the knowledge and skills acquired through training. (P4).*

To address this limitation, a paramedic participant recommended integrating simulated scenarios into the training process. These simulations would provide hands-on experience and enhance decision-making skills in real-time, high-pressure situations. The participant recommended:

*Training should include practical simulations, not just lectures. These simulations could mimic real-life situations like navigating traffic during ambulance journeys, helping paramedics make critical decisions without compromising patient care. (P12).*

In addition to the training format, another key issue raised was the importance of understanding Hajj-specific protocols. Three physician participants emphasised that familiarity with these protocols leads to faster decision-making and more efficient patient care, ultimately saving time and improving outcomes. As two participants explained:

*If I am familiar with the patient’s condition and the required protocols, I can administer necessary medications and arrange for transfer to a specialised facility efficiently. (P10).*

*Understanding prehospital trauma protocols is essential for providing timely and appropriate care in emergencies. (P8).*

However, several paramedic and nursing participants noted that readiness for the Hajj is not solely dependent on protocols or institutional procedures. They highlighted the importance of individual physical and mental preparedness. While most were aware of the protocols, they emphasised that personal resilience was crucial in managing the demanding nature of the event. As participants noted:

*The readiness of the emergency response team depends on the personal readiness of each member. (P13).*

*Knowing the procedures is one thing but being physically fit and mentally prepared for the long hours and high-pressure situations is just as critical. If a responder is exhausted or unprepared, it affects the entire team’s performance. (P6).*

Finally, physician participants underscored the role of continuous self-development as part of professional preparedness. They stressed the need for improvement not only in clinical skills but also in administrative and technological competencies. This broader skillset was viewed as essential for fulfilling their roles effectively during both emergency and routine operations. One participant explained:

*Many areas require self-improvement, not just for me but likely for most colleagues. This includes enhancing medical skills in both emergency and non-emergency contexts, as well as non-medical skills like administrative abilities and computer proficiency. (P9).*

## Crisis management challenges

This theme addressed challenges related to communication, leadership, and coordination in emergency services, highlighting the importance of teamwork and collaboration.

Paramedic participants emphasised that there was strong communication between the Red Crescent and hospitals regarding patient handovers. The ER doctor plays a key role in this process, taking direct responsibility for receiving the patient from the paramedic. The paramedic provides a thorough handover, which includes the patient’s medical history and details of any interventions performed. After receiving this comprehensive handover, the ER doctor promptly signs off, ensuring a smooth transition of care. As the participant noted:

*If you go to the neighbouring hospital, you will find a different policy there, where everything will be taken from you, including medication, history, and procedures (P15).*

However, one paramedic participant expressed frustration over the challenges encountered during patient handovers, despite the availability of beds. The primary issue stemmed from a breakdown in communication between the Red Crescent and the emergency technical director, which negatively impacted patient care. Additionally, there was a notable lack of thoroughness in the handover process by ER staff, further compounding the problem. One participant observed,

*Inconsistencies in patient handovers lead to weak reception at times*,* even when beds are available. There were cases where we had a patient waiting in the ambulance for too long because the receiving team was not properly informed. Sometimes*,* key details about the patient’s condition were missing*,* making it difficult for us to act quickly. The lack of clear and structured communication made an already stressful situation worse. *(P17).

During disaster situations, nursing participants emphasised the critical need for clear leadership to prevent confusion and ensure effective emergency response. They observed that overlapping roles frequently led to inefficiencies in disaster response, often due to the presence of multiple incident commanders. As one participant stated:

*When multiple agencies are involved in a disaster response*,* miscommunication becomes a significant issue. It often happens when each agency or team member wants to assume the role of incident commander*,* leading to confusion about decision-making and authority. This overlap in leadership roles can cause delays*,* disrupt the flow of operations*,* and ultimately hinder the effectiveness of the response. (P3)*.

Physician participants further reinforced the importance of strong, decisive leadership. They emphasised that in rapidly escalating disasters, leaders must give concise, focused, and actionable directions to ensure emergency teams respond swiftly and effectively. Without this, the entire operation risks being compromised, staff may become overwhelmed, resources could be mismanaged, and patient care may deteriorate, leading to preventable deaths. As one participant stated:

*Imagine if leadership is weak or unclear—everything falls apart. The staff gets tired, resources run out, and, ultimately, patients die. (P8).*

In addition to leadership, paramedic participants discussed the immense pressure associated with coordinating healthcare services during Hajj, a large-scale event that accommodates approximately 2,000,000 Pilgrims. Coordinating efforts between healthcare professionals in and out of hospitals in such circumstances can become overwhelming, leading to confusion sometimes. As one participant explained:

*There’s a lot of pressure in coordinating with the Ministry of Health to handle the Hajj movement. I mean*,* I’m not saying everything is lost*,* but there’s confusion. *(P10).

Furthermore, nursing participants stressed the importance of documentation and learning from previous errors to improve future responses. Participants mentioned that by documenting weaknesses and using this information to develop more robust contingency plans, healthcare professionals can better prepare for future disasters. As one stated:

*The key is to acknowledge and address weaknesses*,* utilizing past mistakes to formulate effective plans*,* including contingency plans like Plan ABC. (P3).*

One common example shared by paramedics was the occurrence of errors in incident reporting. These errors often hindered the overall management of emergencies, delaying critical decisions. Physician participants highlighted the need for more accurate and reliable reporting mechanisms to ensure that appropriate actions are taken during a crisis, emphasising that improving reporting is a key component of future contingency planning.

*We have many challenges in our management. Sometimes there are errors in reporting incidents (P7).*

*Sometimes the information we receive is incomplete or incorrect, which affects how we respond. It slows things down and can create confusion during emergencies. (P12).*

In terms of tools and systems, nursing participants noted the extensive reliance on electronic communication systems throughout the stampeded disasters. While these systems generally function smoothly, occasional malfunctions occur, often due to the high volume of calls and system pressure. Despite these challenges, participants acknowledged the significant role technology plays in supporting the hospital’s operations and facilitating timely coordination during emergencies. Participants suggested that further exploration of technological solutions could help address communication challenges.

*I would suggest exploring the utilisation of technology to address the challenges we currently face*,* such as quick responses to ease communication. (P2).*

Finally, paramedic and physician participants emphasised the potential for improving training methods and integrating more advanced technological tools to enhance efficiency. The growing trend of leveraging artificial intelligence (AI) in healthcare was noted as a significant opportunity. By incorporating AI into emergency medical services, hospitals could potentially improve responsiveness and streamline operations, ultimately optimising patient care during critical situations. As one stated:

*Using artificial intelligence (AI) could significantly enhance emergency medical services. (P13).*

## Challenges in prehospital care and patient management

This theme explores the challenges faced by professionals during mass gatherings, focusing on patient access, patient transport, patient care, decision-making complexities, and patient and care management.

### Access and transport

Three paramedic participants shared that there was difficulty in accessing and transporting patients, particularly those located in hard-to-reach areas like high floors in hotels. The participants indicated that providing cardiac care, such as chest compressions, while simultaneously moving a patient from a 48th-floor room to an ambulance presents a critical challenge. As one participant noted:

*Sometimes*,* how I cannot get that patient from this floor to the ambulance while I’m providing care*,* like chest compression*,* is very crucial for that kind of situation. (P14).*

*We often face problems when patients are in high-rise buildings. There’s no time to wait for elevators and carrying equipment while trying to stabilise the patient is extremely difficult. (P16).*

In addition to building access, two paramedic participants pointed out that accessing patients during mass gatherings, such as Hajj, was particularly challenging due to heavy traffic congestion and large crowds, which often delayed timely responses to emergency calls. One participant emphasised this point, stating:

*The most challenges that I faced during that time were the accessibility to the casualties or the patients because of the nature of mass gathering. (P15).*

Navigating holy sites such as Mina and Arafat presents unique challenges for ambulance crews due to the complexity of road regulations and the frequent closure of routes. Participants emphasised the importance of staff developing strong navigational skills and becoming familiar with the road signs and numbers in the holy places to ensure they can respond to emergencies effectively. As one shared:

*This is one of the challenges we faced*,* that people aren’t familiar with the regulations of roads and traffic. (P9).*

However, all participants suggested that relying solely on digital navigation tools, such as Google Maps, may not always be the most efficient way to reach patients in high-pressure environments like Hajj, where roads are often blocked by police or crowded with pilgrims. To address this, participants proposed collaborating with traffic police or municipal authorities to receive real-time updates on traffic conditions, particularly in congested areas. Participants assume this collaboration would help emergency teams navigate more efficiently and provide faster care during large events, ensuring timely response in critical situations.

*Relying solely on Google Maps for navigation may not always lead to the quickest route to reach a patient. (P17).*

### Patient care challenges

Cultural and communication considerations also influenced patient care. Physician participants noted that treating patients from certain nationalities, such as Pakistanis or Indians, often requires heightened caution. When responding to calls, they mentioned that patients sometimes do not explain their symptoms clearly, requiring healthcare professionals to dig deeper to fully understand the patient’s condition. This additional level of care is not based on medical biases but stems from experience with specific cultural or communication barriers that can affect treatment outcomes. As one stated:

*A patient experiencing an asthma attack*,* where a more cautious approach led to the discovery of a serious underlying condition after conducting an ECG. (P8).*

Another significant challenge identified was the fatigue experienced by paramedic participants during responses, particularly when patients became agitated. This agitation often stemmed from misunderstandings of the emergency responders’ role. Some patients assumed the sole responsibility of the emergency staff was to transport them immediately to the hospital, without recognising the value of on-site care. This misunderstanding, participants explained, could hinder treatment. As one noted:

*Some patients will get agitated… they think your job is just to take them to the hospital without giving any kind of care. (P13).*

Building on this, several paramedic participants described disruptions caused by multiple relatives crowding around the patient during assessment and treatment. These relatives sometimes made unrelated requests—such as checking their own blood pressure—which distracted responders from the immediate medical needs of the patient. As participants recalled:

*When providing care to a patient*,* multiple relatives sometimes crowd around and keep talking*,* which makes it difficult to give proper attention to the patient. (P14).*

*During a cardiac arrest case*,* the patient’s family gathered around*,* crying and asking questions while we were performing CPR. Some even tried to touch the patient*,* making it difficult for us to do our job. These situations add unnecessary pressure and can compromise the quality of care we provide. (P15).*

### Clinical management and decision-making

Nursing participants reported encountering a wide variety of health conditions during Hajj, with heatstroke and cardiac issues being the most common. Respiratory conditions, such as distress and bronchial asthma, were also prevalent, driven by the strenuous physical activity and harsh environmental factors. Additionally, occasional cases of infectious diseases. One shared:

*The most common cases we received were heatstroke*,* cardiac cases with various diagnoses*,* respiratory distress*,* bronchial asthma*,* and occasionally infectious diseases. (P2).*

Reflecting on their experiences, participants identified knowledge gaps in handling specific medical cases, particularly burn injuries. One participant acknowledged having only a basic understanding of burn management, emphasising the need for targeted education to prepare healthcare professionals for such complex scenarios. One stated:

*At that time*,* none of us had much knowledge about burns. I had basic knowledge. (P5).*

Adding to this, two nursing participants also described the emotional and physical challenges of treating burn patients, emphasising the severe pain these patients endure. The intensity of their reactions to touch made the process even more demanding for healthcare providers. One participant shared:

*It’s not easy to deal with because the patients are in pain*,* and if you touch them*,* they scream or shout. (P1).*

Environmental and physical demands during Hajj were also significant contributors to medical emergencies. Some physician and paramedic participants noted that long periods of walking under the scorching sun worsened existing health problems and caused new ones, such as dehydration and heatstroke. These conditions placed additional pressure on healthcare teams, who had to manage a high volume of cases. As one participant explained:

*The Hajj journey involves walking more than 15 kilometres and staying outside the camp for over six hours. (P9).*

The challenges extended beyond medical management to include ethical dilemmas and patient compliance issues. Many pilgrims were determined to continue their rituals, even against medical advice. This created tension for healthcare professionals, who had to navigate these desires while upholding their professional responsibilities. As one physician participant explained:

*Sometimes the patients themselves are difficult to deal with*,* either due to language barriers or their desire to leave the hospital and continue their Hajj. This presents ethical considerations—should they complete Hajj or stay in the hospital. (P7).*

Finally, all participants emphasised the importance of careful coordination in tracking bed availability and communicating capacity updates to manage incoming cases. However, despite these efforts, occasional miscommunications sometimes led to delays in patient admissions and operational inefficiencies, complicating the overall management process. One shared:

*We carefully track our available beds and communicate the capacity to incoming cases*,* but sometimes there are miscommunications. (P4).*

## Cultural competence and workforce resilience

Physician participants emphasised several cultural and social factors that significantly impact the delivery of care during the Hajj season. These include language barriers, cultural sensitivities in patient care, the role of female volunteers, and the importance of workforce well-being practices in adapting to the unique demands of Hajj.

Language barriers emerged as a major challenge for emergency responders, given the wide range of nationalities present. Paramedic participants reported that these communication difficulties often hindered their ability to provide timely and effective care. As one explained:

*There are multiple language barriers from different nationalities, affecting how you approach and communicate with the patient. (P15).*

Cultural differences also played a significant role in patient care, particularly with elderly or critically ill patients. Nursing participants described situations where certain examinations were declined due to modesty concerns. For example, performing an ECG or exposing a female patient’s chest for assessment could be refused:

*Sometimes when you encounter a critically ill elderly female patient, they won’t allow you to do an ECG or expose the chest for examination. (P1).*

To address these cultural sensitivities, physician participants pointed to the involvement of female volunteers, including RNs, paramedics, and physicians, who play an essential role in providing care to female patients. These volunteers are often called upon to perform procedures that male staff may not be permitted to do due to cultural restrictions. One participant explained:

*We have female volunteers in our agency, and they can help us perform procedures when cultural restrictions prevent male responders from doing so. (P8).*

In addition to cultural considerations, all participants emphasised the importance of well-being and self-care practices for the emergency workforce, especially when dealing with the heavy workload and high volume of patients. Adequate rest and proper nutrition were critical for maintaining the physical and mental stamina required for effective performance in high-pressure situations. As one participant noted:

*Getting sufficient sleep is crucial, especially when facing the overwhelming nature of work with a high volume of patients and calls to respond to. (P7).*

Beyond rest, some paramedic and physician participants also emphasised the importance of engaging in leisure activities to unwind and restore balance. Spending time with friends or pursuing hobbies, such as playing video games, helps responders relax and maintain a healthy work-life balance. One participant mentioned:

*During challenges, we laugh, change our mood through laughter, creating a jovial atmosphere. (P3).*

## Discussion

This study explored the preparedness and experiences of healthcare professionals, including nursing educators, paramedics, and physicians, during the Hajj mass gatherings, revealing five interconnected themes that highlight the operational, educational, and cultural complexities of delivering care in mass gatherings. The findings shed light on key dimensions of healthcare provision in this uniquely complex and high-stakes environment, identifying opportunities to improve future preparedness, enhance training programmes, and inform policy development.

A central finding was the gap between existing training practices and the realities faced by frontline professionals during Hajj. Although disaster preparedness is increasingly included in health curricula, participants reported limited ability to apply this knowledge, especially in rare and high-risk situations such as chemical exposures and burn injuries. This aligns with previous studies [11–14], which found that theoretical knowledge alone does not equip healthcare professionals for the practical and emotional demands of disaster care. Study participants stressed the need for simulation-based education tailored to the Hajj context, noting that one-off training sessions are insufficient to build and sustain readiness in such extreme environments. These findings support calls for longitudinal, scenario-specific training embedded within continuing professional development across all healthcare disciplines. This recognition of training gaps naturally raises the question of how prepared professionals function in real-time crises, where knowledge alone may not suffice.

Building on the need for better-prepared healthcare professionals, the findings showed that readiness alone is insufficient without clear and coordinated action during crises in the Hajj. Communication and coordination emerged as both critical and vulnerable during the Hajj. Although interagency communication was sometimes effective, care delivery was often disrupted by inconsistent patient handovers, conflicting leadership roles, and the absence of unified protocols. This echoes previous research stressing the importance of role clarity and structured command systems in mass casualty events [15–16]. Notably, overlapping leadership during emergencies created tensions that not only delayed action, it eroded team confidence. From an educational perspective, these findings highlight the value of integrating crisis communication and leadership training into clinical education, equipping nursing students and other healthcare professionals to function effectively within complex hierarchical systems during disaster scenarios [17]. Understanding these coordination challenges also requires attention to the operational realities that frontline professionals face, which further complicate effective response.

These coordination challenges were often intensified by the operational realities of delivering prehospital care during Hajj. Patient access and transport were hindered by obstructed roads, high-rise buildings, and dense crowds—often while lifesaving interventions were in progress. While disaster medicine research has typically focused on in-hospital logistics [18–20], our findings highlight the under-recognised burden of navigating these field-based obstacles in real time. Such barriers point to the need for expanded paramedicine and nursing curricula that address dynamic triage, improvisational care strategies, and field-based clinical decision-making. Additionally, participants’ experiences with family interference and cultural misunderstandings emphasise that interpersonal and community communication skills are as essential as technical expertise. These operational and interpersonal challenges, in turn, intersect with ethical and cultural considerations that further shape care delivery during Hajj. These operational and interpersonal challenges, in turn, intersect with ethical and cultural considerations that further shape care delivery during Hajj.

Beyond the logistical and clinical dimensions, delivering care during Hajj also revealed a layer of ethical and cultural complexity. The high incidence of heatstroke, cardiac conditions, and respiratory distress aligns with prior work on health risks in extreme climates [21, 22], with findings from this current study adding a further dimension by showing how patient refusal of treatment, often to continue religious rituals, can place professionals in ethical dilemmas. Moreover, language barriers and cultural sensitivities, already well-documented in global health literature [23, 24], were reported here as directly affecting the delivery of urgent care. This highlights the critical role of both cultural competence alongside clinical expertise. The findings also reinforce the importance of workforce diversity, including the strategic deployment of female responders in contexts where cultural norms restrict the involvement of male clinicians. Taken together, these layers of complexity underscore that preparedness extends beyond clinical knowledge to include leadership, communication, and cultural sensitivity.

Taken together, this study reveals that preparedness for healthcare professionals in the Hajj context is multifaceted, requiring not only clinical knowledge and procedural readiness but also strong leadership, cultural competence, adaptive communication, and emotional resilience. These findings offer new insights into how nurse educators and healthcare systems can collaborate to prepare frontline professionals for complex mass gatherings. Adopting a systems-based approach that integrates context-specific training, interprofessional coordination, and technological literacy is essential to enhancing care delivery, workforce sustainability and resilience in disaster settings [3, 25].

### Theoretical implications

Systems Theory provides a framework for understanding complex situations by examining the dynamic interactions among individuals, teams, organisations, and policy environments. It emphasises how components of a system influence one another, enabling the identification of strengths, vulnerabilities, and potential leverage points to enhance performance and resilience [26].

In this study, Systems Theory was applied as an **interpretive lens** to contextualise the relationships between the inductively generated themes, rather than as a prescriptive model guiding analysis. The five themes illustrate how disruptions in one domain—such as communication breakdowns or leadership ambiguity—can propagate across the healthcare system and influence care delivery during the Hajj.

Importantly, the findings also extend conventional systems models by highlighting dimensions that are often underrepresented, particularly socio-cultural and religious influences. Within the Hajj context, cultural sensitivities, religious obligations, and pilgrim expectations significantly shape clinical decision-making, professional roles, and system performance. These insights suggest the need for a more culturally adaptive application of Systems Theory—one that integrates both structural elements (such as governance and coordination) and symbolic dimensions (such as beliefs, values, and religious practices) when examining healthcare delivery in religious mass-gathering settings.

### Policy and practice implications

To improve disaster response and resilience during Hajj, a national health strategy should be developed through multi-agency collaboration and reviewed annually. This should include protocols for vaccination, surveillance, emergency preparedness, and healthcare workforce planning. International coordination with Hajj-sending countries through shared digital platforms and bilateral agreements can enhance data-sharing and public health surveillance. Frontline professionals, especially nurses and paramedics, should be actively involved in planning to ensure policies reflect operational realities. Improvements in working conditions, mental health support, and authorised decision-making at the point of care are essential to enhance service delivery and staff well-being. Real-time crowd monitoring, digital command systems, and drones can further strengthen coordinated responses in high-risk zones.

### Implications for future research

Future research that employs mixed-method approaches, larger and more diverse sample sizes, or longitudinal designs could strengthen the transferability of findings and provide a more comprehensive understanding of disaster preparedness, interprofessional coordination, and workforce resilience in mass-gathering healthcare contexts. Given that the qualitative design used in this study limits transferability, readers should consider the unique cultural, logistical, religious, and operational context of the Hajj when applying these insights elsewhere or to other mass gatherings. Further studies should also examine the effectiveness of current training interventions, the long-term impact of Hajj participation on professional development, and the role of technology in addressing communication and coordination challenges.

### Strengths and limitations

One of the strengths of the current study is the aggregation of data across various healthcare fields, including physicians, paramedics and nursing educators who participated in the Hajj. Aggregating data from diverse HCPs provides a more comprehensive understanding of HCPs’ experiences during the Hajj. While this study offers rich insights, it is important to acknowledge its limitations. As this study is qualitative, the findings may not be directly transferable to other settings. The unique cultural, religious, logistical, and operational characteristics of the Hajj context shape participants’ experiences, so readers should interpret and apply these insights with caution when considering different healthcare environments. Additionally, participants’ recollections may be influenced by time and personal perception, potentially introducing bias.

## Conclusion

This study explored the preparedness and experiences of nursing educators, paramedics, and physicians during the Hajj pilgrimage, revealing critical insights into the challenges of delivering care in a complex, high-pressure mass gathering. The findings highlight gaps in disaster preparedness, inconsistent clinical protocols, communication breakdowns, and the need for greater cultural competence. They also underscore the importance of simulation-based training, interdisciplinary coordination, and workforce well-being in ensuring an effective healthcare response. To enhance future preparedness, healthcare systems and educational institutions must adopt integrated, context-specific training and policy strategies that equip professionals with the skills, resilience, and cultural awareness needed to provide safe and effective care during large-scale events like the Hajj.

## Supplementary Information

Below is the link to the electronic supplementary material.

Supplementary Material 1

## Data Availability

The datasets generated and/or analysed during the current study (interview transcripts) are not publicly available due to confidentiality and ethical restrictions but are available from the corresponding author on reasonable request.
